# Dual-camera design for hyperspectral and panchromatic imaging, using a wedge shaped liquid crystal as a spectral multiplexer

**DOI:** 10.1038/s41598-020-60413-8

**Published:** 2020-02-26

**Authors:** Shauli Shmilovich, Yaniv Oiknine, Marwan AbuLeil, Ibrahim Abdulhalim, Dan G. Blumberg, Adrian Stern

**Affiliations:** 1 Department of Electrical and Computer Engineering, School of Electrical and Computer Engineering, Ben-Gurion University of the Negev, P.O.B. 653, Beer-Sheva, 8410501 Israel; 2 Department of Electro-Optics and Photonics Engineering, School of Electrical and Computer Engineering, Ben-Gurion University of the Negev, P.O.B. 653, Beer-Sheva, 8410501 Israel; 3 The Ilse Katz Institute for Nanoscale Science and Technology, Ben-Gurion University of the Negev, P.O.B. 653, Beer-Sheva, 8410501 Israel; 4 Department of Geography and Environmental Development, Ben-Gurion University of the Negev, P.O.B. 653, Beer-Sheva, 8410501 Israel

**Keywords:** Optical sensors, Optoelectronic devices and components, Liquid crystals, Imaging and sensing, Optical spectroscopy

## Abstract

In this paper, we present a new hyperspectral compact camera which is designed to have high spatial and spectral resolutions, to be vibrations tolerant, and to achieve state-of-the-art high optical throughput values compared to existing nanosatellite hyperspectral imaging payloads with space heritage. These properties make it perfect for airborne and spaceborne remote sensing tasks. The camera has both hyperspectral and panchromatic imaging capabilities, achieved by employing a wedge-shaped liquid crystal cell together with computational image processing. The hyperspectral images are acquired through passive along-track spatial scanning when no voltage is applied to the cell, and the panchromatic images are quickly acquired in a single snapshot at a high signal-to-noise ratio when the cell is voltage driven.

## Introduction

Over the last decades, spectral imaging^1–4^ has become progressively utilized in airborne and spaceborne remote sensing tasks^5,6^, among many others. Today it is used in many fields, such as vegetation science^7^, urban mapping^8^, geology^9^, mineralogy^10^, mine detection^11^ and more. A large amount of these spectral imagers take advantage of the platform’s motion, and acquire the spectral data by performing along-track spatial scanning of the imaged object^4,12^. The spectral information could then be measured directly, that is, by firstly splitting it into spectral components using a dispersive or diffractive optical element, followed by a direct measurement of each component. Another approach is to acquire the spectral information indirectly, incorporating multiplexed and coded spectral measurements. This allows to benefit from Fellgett’s multiplex advantage^13,14^, and achieve a significant gain in optical throughput, at the cost of less intuitive system design and the need for post-processing. A classic example of indirect spectral acquisition is Fourier transform spectroscopy, which is used both for spectrometry^15^ and spectral imaging (e.g.^16,17^). As conventional Fourier transform spectroscopy systems are based on mechanically scanned Michelson or Mach-Zehnder interferometers, they demand strict stability and precision requirements. Thus, in harsh aerial and space environments, significant sophistication in their mechanical construction is required, and high-cost translational stages are incorporated to preserve the interferometric stability^18,19^. Indirect spectral acquisition can also be achieved by utilizing voltage driven liquid crystal phase retarders^20–28^, evading the need for incorporating moving parts which result in these strict stability requirements. One such system, which was recently presented by our group, is the Compressive Sensing Miniature Ultra-Spectral Imaging (CS-MUSI) system^24,25,27^. It performs spectral multiplexing by applying various voltages to a thick liquid crystal cell (LCC), thus achieving distinctive wideband spectral encodings. Another system, which is described in^26^, alternatively performs spectral multiplexing by cascading several voltage driven LCCs.

In this work, we present a camera designed for dual operational modes; hyperspectral (HS) and panchromatic imaging. The camera utilizes a wedge shaped LCC as a spectral multiplexer and therefore consists of no moving parts. Hence, when compared to the previously mentioned mechanically scanned interferometers, it is significantly more tolerant to vibrations, and no special sophistication is required in its mechanical construction. Yet, as in other airborne and spaceborne cameras, system vibrations could cause blur effects and a degradation in image quality^29–31^. Compared to our CS-MUSI system, the presented camera also enjoys the advantage of passively acquiring the HS image, which is a key property in spaceborne and unmanned airborne tasks. Instead of actively applying voltages to the LCC to achieve different spectral encodings for the HS imaging process, the system passively encodes the spectrum through along-track scanning, by taking advantage of the motion of an airborne or spaceborne platform, and the wedge shape of the LCC. As the system performs spectral multiplexing using its LCC, it also benefits from Fellgett’s multiplex advantage and has a high optical throughput, which is very important in low photon-flux situations such as spaceborne tasks. Apart from the ability to passively acquire a HS image, the system transforms into a panchromatic snapshot camera when a voltage is shortly applied to its LCC. The applied voltage alters the LCC’s spectral transmission (ST) to allow the energy in all the desired wavelength range to pass through the cell. This allows to quickly obtain the spatial information of the imaged scene in a single snapshot, hence it can be operated at high frame rates and with high signal-to-noise ratio (SNR). The described duality permits installing a single camera on the airborne or spaceborne platform, to perform both HS and panchromatic tasks, instead of two separate cameras. Therefore, it allows the use of a larger aperture for the single camera, which results in both a higher optical throughput, and improved optical spatial resolution. In addition, the spectral multiplexing achieved by the LCC is performed in the spectral domain, that is, without performing any optical spectral-to-spatial transformations for spectral acquisition. Therefore, there is no tradeoff between the spatial and spectral resolutions, and the spatial resolution achieved in the HS image is identical to the high spatial resolution achieved in the panchromatic image, which is determined by the chosen optics and the sensor array solely.

## The Wedge Shaped LCC’s Spectral Attributes

**Figure 1:**
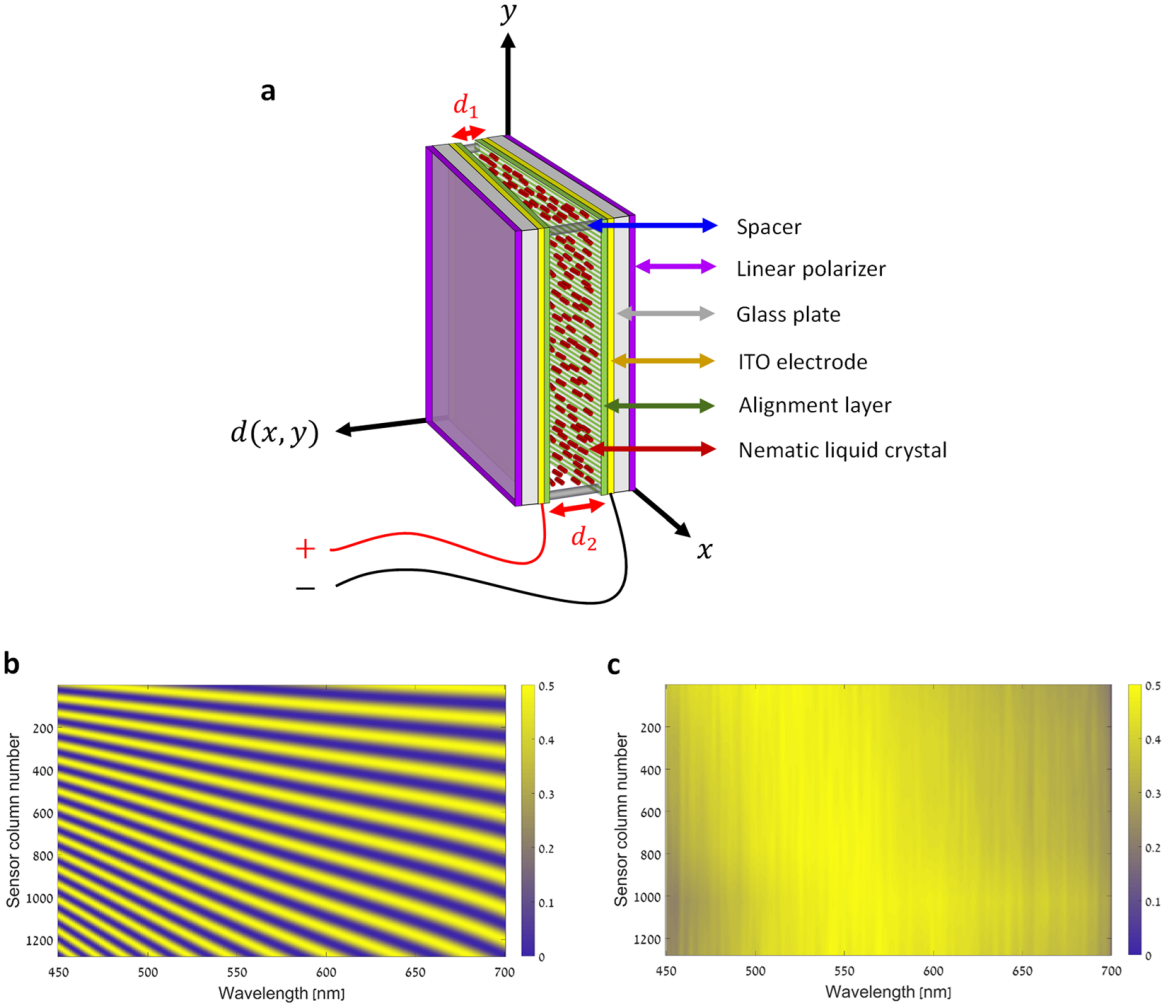
The wedge shaped LCC and the system’s measured ST maps, at a single row of pixels, for each of the dual HS-panchromatic operational modes. (**a**) An illustration of the geometry of the wedge shaped LCC. The *x* and *y* axes are defined so that the cell gap, *d*(*x*, *y*), changes only along the $x$ direction, as emphasized by *d*_1_ and *d*_2_. For a detailed description of the LCC’s layers and fabrication process see Methods – LCC device. (**b**) The ST with no voltage applied to the LCC. Different oscillatory behaviors are obtained at each column due to the linearly increasing cell gap of the LCC. (**c**) The ST of the voltage driven LCC which is used for panchromatic imaging. The ST is approximately constant at all columns and wavelengths.

The main optical component of the imager presented in this work is its wedge shaped LCC (Fig. 1a). Such a configuration for LCCs has been previously used in several studies for various applications. These include tunable angular shearing interferometer^32^, depolarizer based Cholesteric Liquid Crystal (CLC)^33^ and continuous spatial tuning of laser emissions based dye-doped CLC^34^. In this work, the wedge shaped LCC allows to perform both the necessary spectral multiplexing for the HS image acquisition during the along-track scanning, and to acquire a panchromatic image in one snapshot when a voltage is applied to it. The LCC is located in the image plane, and is optically conjugated to the imager’s two-dimensional (2D) sensor array using a 1:1 relay lens (Methods – Experimental optical setup). Therefore, each point in the imaged scene is spectrally encoded as it passes through the LCC and captured by the 2D sensor array. The spectral encoding that corresponds to each sensor pixel is determined by the ST of the LCC at the matching location. The ST of the wedge shaped LCC, when inserted between two linear crossed polarizers (Methods – LCC device), is given by^35,36^

1 $$\begin{array}{c}T(V,\lambda ;x,y)\propto \frac{1}{2}-\frac{1}{2}\,\cos (\frac{2\pi \Delta {\rm{n}}(\lambda ,V)d(x,y)}{\lambda }),\\ d(x,y)=ax+by+c.\end{array}$$

here, *λ* is the wavelength, *V* is the voltage applied to the LCC, $\Delta {\rm{n}}(\lambda ,V)$ is the effective birefringence, *x* and *y* are spatial coordinates, and *d*(*x*, *y*) is the spatially varying cell gap which has a planar profile. By defining the *x* and *y* axes as shown in Fig. 1a, the parameter *b* can be set to zero, meaning that the cell’s gap, *d*(*x*, *y*), linearly varies along the *x* direction solely, and can be denoted as *d*(*x*) = *ax* + *c*. As the LCC is placed in the image plane so that the defined $x$ axis is aligned with the rows of the 2D sensor array, and the defined $y$ axis is aligned with its columns (Methods – Experimental optical setup), the cell gap is approximately constant per column of pixels and varies from column to column, that is, it varies along each single row of pixels. From Eq. (1) and owing to the alignment and optical conjugation of the LCC and the sensor, we can deduce that the ST of the system is identical at pixels located in the same column of the sensor, but varies along pixels located in the same row. Therefore, the ST is identical at each row of pixels and can be fully characterized by measuring it at a single row (Fig. 1b). As explained in the Image acquisition section, the oscillatory behavior of the ST over a broad wavelength range, which varies along the sensor’s columns (Fig. 1b), allows capturing multiple different spectrally encoded images of the imaged scene. The spectra of the imaged scene at each spatial pixel can then be computationally decoded, as demonstrated in the Results section. Figure 1b shows the measured ST of one row of pixels, and its measurement process is explained in the Methods – Calibration section. When a suitable high voltage is applied to the LCC, its effective birefringence decreases, causing the argument of the cosine to reach a value close to *π* with low dispersion in the visible range. This results in a slow oscillatory behavior of the LCC’s ST. One can also operate the device between parallel polarizers and obtain a flat maximum ST at a high enough voltage. However, practical realization of such a solution requires high quality polarizers in order to avoid degradation of the modulation depth at zero voltage. Therefore in our demonstration we chose to use the crossed polarizers scheme. The ST can then be considered to be approximately constant, allowing the energy at all wavelengths and locations to pass through the cell equally, and can therefore be used to acquire a panchromatic image in one snapshot. Figure 1c shows the measured ST in the case of a voltage driven LCC.

## Image Acquisition

**Figure 2:**
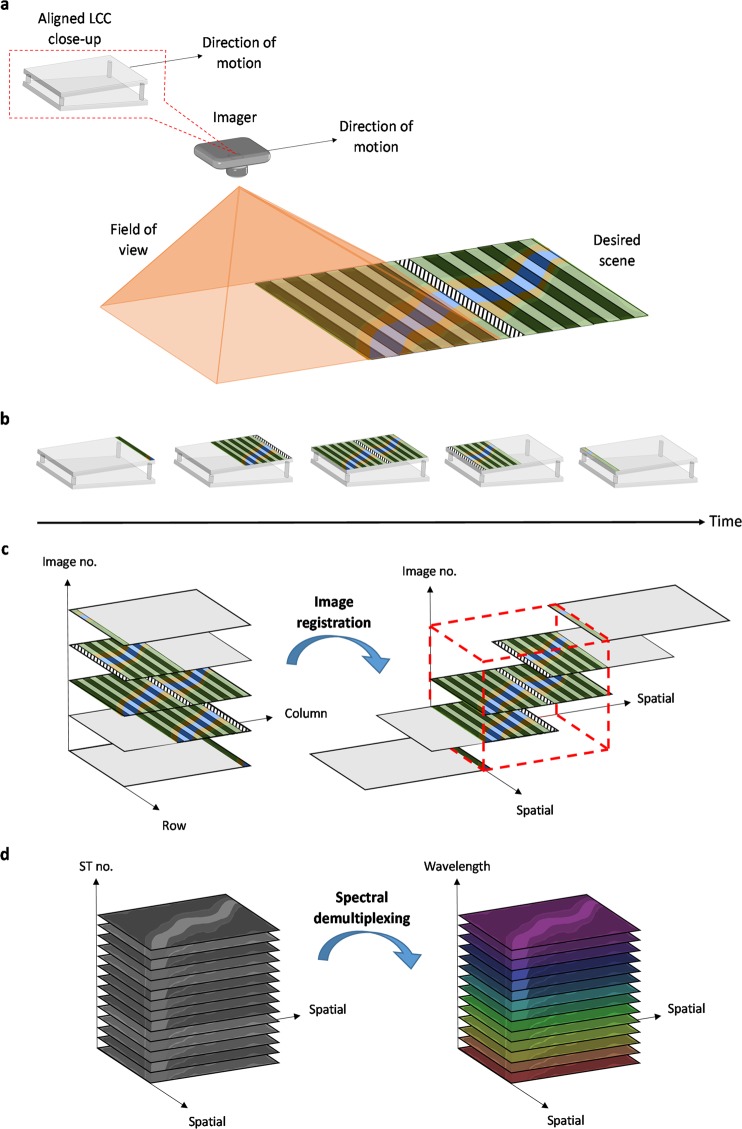
HS image acquisition process. (**a**) The imager scans the desired scene during its motion. The LCC is aligned with the direction of motion to perform different spectral encoding for each strip of the imaged scene. (**b**) The imaged scene gradually travels along the LCC over time, allowing recording differently spectrally multiplexed measurements of each strip of the scene. (**c**) The set of acquired images (left) is registered and aligned (right) according to the spatial context of the imaged scene, which is marked by a dashed red outline of a cube. (**d**) Through spectral demultiplexing, a HS data-cube of the imaged scene (right) is reconstructed from the acquired spectrally multiplexed HS data-cube (left) of the scene.

The image acquisition process depends on whether the desired image type is HS or panchromatic. When a HS image is desired, no voltage is applied to the LCC, and the image is passively captured via along-track scanning of the desired scene. As demonstrated in Fig. 2a, the process begins by aiming the imager’s field of view so it passively scans the desired scene during the platform’s motion. Then, the imager is aligned so that the sensor’s columns are oriented along the cross-track direction, and the rows along the along-track direction. As previously explained, because the LCC and the sensor are aligned and optically conjugated, the ST varies from column to column of sensor pixels (Fig. 1b). Since the sensor is pushed along the flight direction at a fixed cross-track angle as the platform moves forward, each strip of the scene, which is oriented along the cross-track direction, is imaged upon a column of pixels. Hence, it is filtered by a certain ST which is identical for all the pixels in that column. This allows acquiring different spectrally multiplexed measurements of each strip of the scene as it is imaged upon the different columns of the sensor during the motion of the platform. Figure 2b demonstrates this process and shows how the strips of the scene are gradually scanned by the system and filtered when imaged upon the LCC. For the clarity of the explanation, we marked one strip of the scene so it is easy to follow its motion upon the LCC as time progresses and the platform moves. At the end of the acquisition process, the recorded images are registered and aligned according to the spatial context of the imaged scene, as shown in Fig. 2c. The image registration can be achieved either by calculating the motion of the strips in the image plane from the geometry of the motion, or by estimating it numerically from the sequence of recorded frames. The registration of the images allows arranging the acquired spatial data of the imaged scene in layers which correspond to the different STs used during the acquisition, as the imaged scene is scanned by each sensor column of pixels. A spectrally multiplexed HS data-cube (Fig. 2d) can therefore be formed, which consists of two dimensions that represent the spatial domain, and one dimension which represents the different STs used during the acquisition. Next, a demultiplexed HS data-cube (Fig. 2d) is reconstructed from the spectrally multiplexed HS data-cube using a sparse recovery solver such as OMP^37^ or TwIST^38^, and a sparsifying transform such as the wavelet transform or a relevant dictionary^39,40^. As shown in Fig. 2d, the demultiplexed HS data-cube consists of two dimensions which represent the spatial domain, and one dimension which represents the spectral domain. Additionally, if a panchromatic image is sought, a voltage is simply applied to the LCC, and the image can be captured in one snapshot as the ST is approximately constant (Fig. 1c).

## Results

To demonstrate the capabilities of the imager presented in this work, an experimental optical setup was assembled (see Methods – Experimental optical setup), containing a wedge shaped LCC which was manufactured in-house. During the system’s calibration process (see Methods – Calibration), the ST was measured at each column of sensor pixels (Fig. 1). Its measurement is crucial as the precise ST is needed for the demultiplexing process, in order to obtain an estimation of the original HS data-cube from the spectrally multiplexed measured data-cube. Additionally, the LCC and the sensor were aligned as explained in the previous section. The results of three experiments and a simulation are presented. In the experiments, both emission and reflection HS and panchromatic imaging were carried out, and airborne HS and panchromatic imaging were simulated. All the HS images were acquired by creating a relative motion between the imager and the imaged objects, to gradually scan the imaged object as explained in Fig. 2. The panchromatic images were acquired in a single snapshot while applying a voltage to the LCC.

### Emission hyperspectral imaging experiments

**Figure 3:**
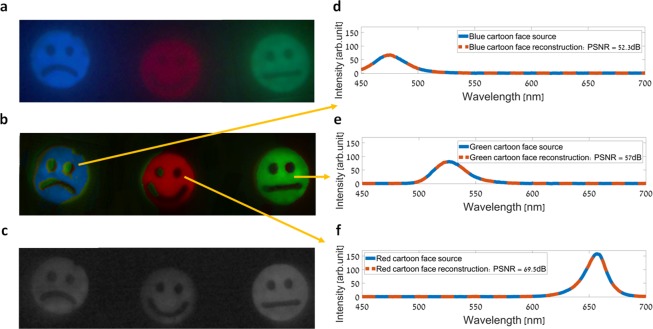
HS imaging of three cartoon faces transilluminated with LED light sources of different colors. (**a**) RGB color image of the objects. The image was captured with a standard RGB color camera. (**b**) RGB representation of the demultiplexed HS image, which was restored from the spectrally multiplexed image captured with our imager. (**c**) The acquired panchromatic image of the objects. (**d**–**f**) The spectra at three points in the demultiplexed HS data-cube (dashed line curves), compared to the spectra of the three respective cartoon faces as measured by a commercial grating based spectrometer (solid line curves).

The imager’s performance is firstly demonstrated in an emission HS imaging scenario. Two such experiments are presented. In the first experiment, three colored LED array light sources with a wide spectral distribution were used (Thorlabs LIU001, LIU002 and LIU003 LED array). The light sources transilluminated three cartoon faces with red, green and blue LED lights, as can be seen in Fig. 3a which was captured by a standard RGB color camera. A HS image was acquired by spatially scanning the objects with our imager. The spatial scanning was achieved by moving the objects relative to the imager, so that the sensor’s columns are oriented along the cross-track direction. A spectrally multiplexed HS data-cube was then obtained by capturing 251 images during the spatial scanning, and performing image registration along a common spatial grid with sub-pixel accuracy using a phase correlation algorithm^41,42^. A demultiplexed HS data-cube consisting of 251 spectral bands in the 450–700 nm wavelength range was then restored (full rank recovery) using the OMP solver^37^ for sparse recovery, and a dictionary^39,40^ as the sparsifying transform. Figure 3b shows a pseudo-color image obtained by projecting the obtained demultiplexed HS datacube onto the RGB color space, which is in great agreement with the ground truth image shown in Fig. 3a. Additionally, a panchromatic image of the objects, which can be seen in Fig. 3c, was acquired while applying a 2 kHz sine waveform voltage with a 40 V amplitude to the LCC. Figure 3d–f show the spectra obtained at three points in the demultiplexed HS data-cube, in comparison to the spectra of the three respective LEDs as measured with a commercial grating based spectrometer. Excellent accuracy is demonstrated, and very high peak signal-to-noise ratio (PSNR) values (52.3–69.5 dB) were obtained comparing the source and demultiplexed spectra curves.

**Figure 4:**
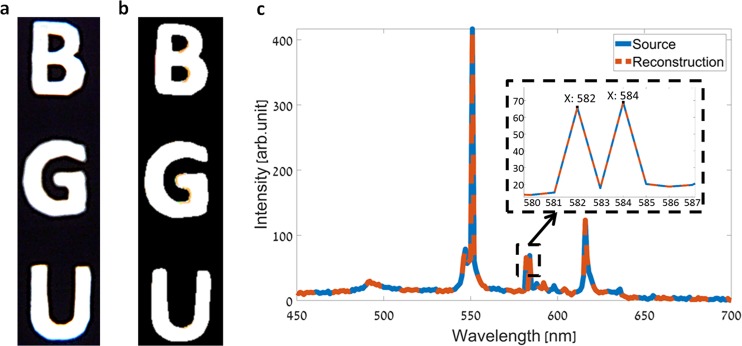
HS imaging of the BGU logo transilluminated with a fluorescent light source. (**a**) RGB color image of the object. The image was captured with a standard RGB color camera. (**b**) RGB representation of the demultiplexed HS image, which was restored from the spectrally multiplexed image captured with our imager. (**c**) The spectrum at an arbitrary point in the demultiplexed HS data-cube of the transilluminated object (red dashed line curve), compared to the spectrum of the fluorescent light source as measured by a commercial grating based spectrometer (blue solid line curve). The close-up of the graph which is shown in the inset, demonstrates the imager’s ability to separate two close spectral lines and illustrates a 2 nm spectral resolution.

In the second demonstrated experiment, a fluorescent light source with a spectral distribution consisting of several narrow spikes was used. The light source transilluminated the Ben-Gurion University (BGU) logo, as can be seen in Fig. 4a which was captured by a standard RGB color camera. As in the previous experiment, a spectrally multiplexed HS data-cube was acquired by moving the objects relative to the imager to perform the spatial scanning, and capturing spectrally multiplexed images. Again, 251 images were captured during the spatial scanning, followed by an image registration process. The demultiplexed HS data-cube, consisting of 251 spectral bands in the 450–700 nm wavelength range, was then restored using the OMP solver, and a dictionary as the sparsifying transform. In a similar manner to the previous experiment, Fig. 4b shows a pseudo-color image obtained by projecting the demultiplexed HS data-cube onto the RGB color space, which is in great agreement with the ground truth image shown in Fig. 4a. In Fig. 4c, the spectrum obtained at a single point in the demultiplexed HS data-cube, is compared to the spectrum of the respective fluorescent spectrum as measured with a commercial grating based spectrometer. Apart from the excellent agreement between the two curves, the inset of Fig. 4c shows a close-up of the spectrum in the 580–586 nm wavelength range, which demonstrates the imager’s spectral resolution and ability to separate two spectral lines that are 2 nm apart from one another.

### Reflection hyperspectral imaging experiment

**Figure 5:**
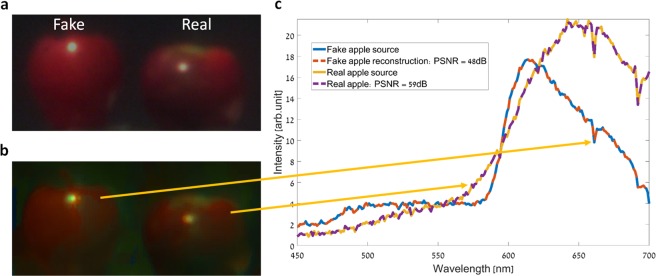
HS imaging of real and fake (plastic) red apples. (**a**) RGB color image of the objects as captured by a standard RGB color camera. (**b**) RGB representation of the demultiplexed HS image, which was restored from the spectrally multiplexed image captured with our imager. (**c**) The spectra at two points in the demultiplexed HS data-cube (dashed line curves), compared to the spectra of the two respective real and fake apples, as measured by a commercial grating based spectrometer (solid line curves).

The second scenario in which the imager’s performance was demonstrated was of reflection HS imaging. In this experiment, two objects were illuminated by sunlight, and the reflected light was captured by the imager. As HS imaging allows to identify different imaged materials and objects by observing their spectra along with the spatial information, the objects were chosen to be a real red apple and a fake (plastic) red apple, which could later be distinguished using the obtained HS image. Figure 5a shows the imaged objects as captured by a standard RGB color camera. The spatial scanning of the object for acquiring the spectrally multiplexed HS data-cube, and the demultiplexing process, were identical to those in the previous experiments. Figure 5b shows a pseudo-color image obtained by projecting the demultiplexed HS datacube onto the RGB color space, which is in great agreement with the ground truth image shown in Fig. 5a. In Fig. 5c, the spectrum obtained at two points in the demultiplexed HS data-cube, is compared to the spectrum of the respective real or fake apple spectra as measured with a commercial grating based spectrometer. Excellent accuracy is demonstrated, and very high reconstruction PSNR values (48 dB, 59 dB) were obtained comparing the source and demultiplexed spectra curves.

### Airborne remote sensing hyperspectral imaging simulation

As push-broom and along-track spatial scanning based imaging methods are naturally used in airborne and spaceborne remote sensing tasks, we decided to demonstrate the performance of the presented imager and the proposed method in such a scenario. Because the imager has not yet been installed on an airborne or spaceborne platform, such as a drone or nano-satellite, the image acquisition and along-track scanning process were simulated. For the simulation, real data from a HS airborne system^43^ was used. The system is loaded with an SIM.GA VNIR sensor which is a push-broom avionic HS instrument manufactured by Selex ES. Its spectral range is 400–1000 nm with spectral sampling of 1.2 nm, providing 512 spectral bands. The sensor has 1024 spatial pixels with an Instantaneous Field of View (IFOV) of 0.499mrad, which give a total Field of View (FOV) of ±15°, and the ground sampling resolution at an altitude of 1200 m is approximately 0.6 m.

**Figure 6:**
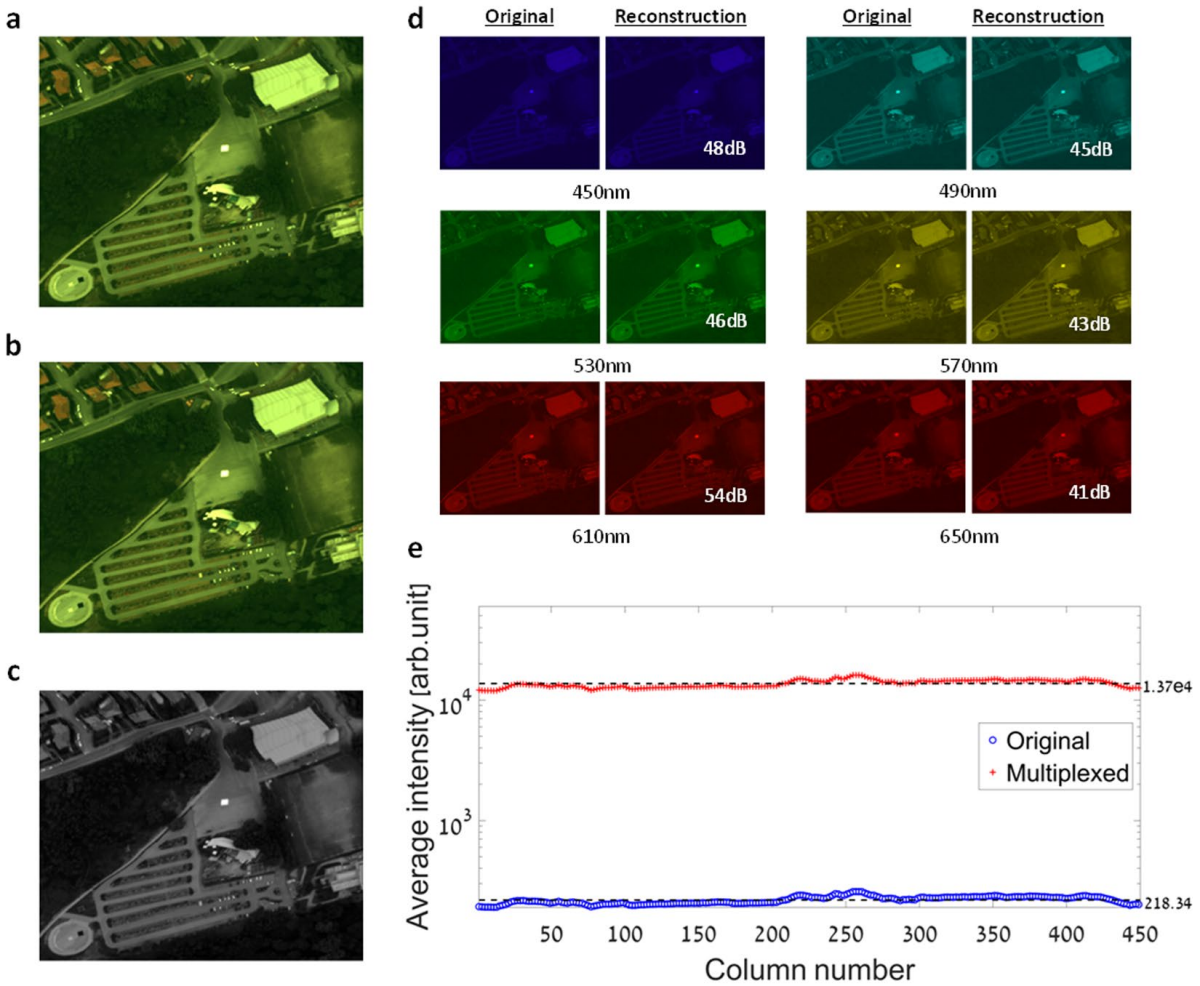
Airborne HS and panchromatic imaging simulation. (**a**) Original RGB color image of the “$\alpha$ scenario”. (**b**) RGB representation of the demultiplexed HS image, which was restored from the simulated spectrally multiplexed image. (**c**) The simulated panchromatic image obtained by spectrally filtering the HS image of the “$\alpha$ scenario”, using the panchromatic ST map (Fig. 1c). (**d**) Six subfigures of spectral channel pairs from the original and demultiplexed HS data-cubes. The wavelength corresponding to each displayed spectral channel (450–650 nm with steps of 40 nm), is located below each pair of images. The high precision obtained in each displayed spectral channel is shown in terms of a PSNR value, which is located at the bottom right of each subfigure. (**e**) A comparison between the average intensity measured by the SIM.GA VNIR sensor (blue circle) and the presented imager (red plus sign), in the airborne remote sensing HS imaging of the “$\alpha$ scenario” simulation. Each plotted value represents the average intensity measured in the corresponding column of spatial pixels, in each of the systems. The average intensity measured by the presented imager across all columns (black dashed line), is approximately sixty times higher than the corresponding value measured by the SIM.GA VNIR sensor.

Out of the airborne HS dataset, the “$\alpha$ scenario” was chosen for the simulation, which shows a parking lot in a suburban vegetated area, as can be seen in the RGB image in Fig. 6a. Next, some pre-processing was performed on the HS “$\alpha$ scenario” image to make it compatible with the measured ST (Fig. 1) of our imager. It included cropping and interpolating the spectral information to match the spectral grid of the ST. The along-track scanning of the scene was then simulated by performing spectrally multiplexed measurements of the scene using 251 different spectral filters. Each filter corresponds to one of the first 251 consecutive rows in the matrix representation of the ST shown in Fig. 1b. The spectrally multiplexed measurements were obtained by an element-wise multiplication of the spectrum of each pixel in each spatial column of the HS “$\alpha$ scenario” image, with each of the first 251 rows of the ST. This yielded a full simulated spectrally multiplexed HS data-cube of the “$\alpha$ scenario”, as would be acquired by the presented imager in similar imaging conditions, using identical aperture size and exposure time. Next, using the TwIST^38^ solver for sparse recovery, and the Daubechies 4 wavelet basis (db4 in MATLAB) as the sparsifying transform, a HS demultiplexed data-cube was restored, consisting of 251 spectral bands in the 450–700 nm spectral range. The simulation result is visualized in Fig. 6. Figure 6b shows a pseudo-color image obtained by projecting the demultiplexed HS data-cube onto the RGB color space. Evidently, there is an excellent agreement between Fig. 6b, and the ground truth shown in Fig. 6a. Additionally, Fig. 6d displays a comparison of six different spectral channels from the original and demultiplexed data-cubes (a full comparison can be seen in the Supplement 1 video). The PSNR achieved in each comparison is shown as well, and an average PSNR of 47 dB was obtained by comparing all 251 spectral channels.

As the presented imager has panchromatic imaging capabilities as well, a simulation was performed to demonstrate the single snapshot image acquisition when a voltage is applied to the LCC, allowing a quick acquisition of the spatial information of the imaged scene with a high SNR. The image acquisition was simulated by filtering the original HS “$\alpha$ scenario” data-cube using the ST map shown in Fig. 1c. Each spatial column in the HS data-cube was filtered using the corresponding row of the ST map. This means that the spectrum vector at each pixel in a spatial column of the HS data-cube, was element-wise multiplied by a spectral filter vector which is the row of the ST map that corresponds to that spatial column. Figure 6c shows the resulting panchromatic image of the “$\alpha$ scenario”.

As emphasized in the Introduction section, the main advantage in performing spectral multiplexing for HS imaging is the benefit of high optical throughput. This can be demonstrated through a comparison between the measured intensity values from the original and simulated spectrally multiplexed HS “$\alpha$ scenario” data-cubes, captured by the push-broom avionic HS instrument and our imager, respectively. As the channels of the original HS data-cube correspond to specific spectral bands, and the channels in the simulated spectrally multiplexed HS data-cube correspond to specific spectral multiplexing filters, an element-wise comparison would be unsuitable. Instead, prior to the comparison the intensity values were averaged through all channels per spatial pixel in both the data-cubes. This resulted in an average value for each spatial pixel in the original HS data-cube, which represents the average intensity measured in the entire 450–700 nm spectral range. In comparison, the average value calculated in the corresponding spatial pixels of the simulated spectrally multiplexed HS data-cube, represents the average intensity measured after filtering the spectrum intercepted at each spatial pixel, using all the different spectral filters. As our imager captures the HS image of the scene column by column of pixels, the average values obtained per spatial pixel were further averaged per each column of spatial pixels. This allows demonstrating the average gain in optical throughput per acquired spatial column in the HS data-cube. The comparison results are presented in Fig. 6e. It shows that an average intensity of 218.34 (arbitrary units) was measured in all the spatial columns of the original HS “$\alpha$ scenario” data-cube. In comparison, the average intensity value measured in all the spatial columns of the simulated spectrally multiplexed HS data-cube was 1.37e4 (arbitrary units), which is approximately two orders of magnitude higher, hence demonstrating the significant gain in optical throughput as a result of the performed spectral multiplexing. An additional visualization of the described comparison can be seen in Supplement 2.

## Discussion

The capabilities of the presented imager were demonstrated in both experiments and a simulation. The emission and reflection imaging experiments have demonstrated the performance of the dual HS-panchromatic operational modes. For the HS mode, excellent precision was shown by comparing the achieved demultiplexed spectra, with corresponding spectra measurements acquired by a commercial grating based spectrometer. Additionally, panchromatic images of the imaged objects were acquired successfully. In the simulation, the high optical throughput of the imager was demonstrated by comparing its average measured intensity values of a remotely sensed scene, to the values measured by a conventional push-broom avionic HS instrument. The simulation also demonstrated the panchromatic imaging capabilities of the imager in airborne imaging tasks.

The significantly high optical throughput of the presented imager is a result of the performed spectral multiplexing. Classical HS imagers which acquire the spectral information of the imaged scene through direct spectral measurements, e.g. narrowband tunable filter based HS imagers^44,45^, allow a small portion of the spectrum at each spatial pixel to pass through the filter. This results in a low optical throughput when compared to HS imagers that perform spectral multiplexing, which allows a large portion of the spectrum to pass through at each pixel at the cost of post-processing for spectral reconstruction. The Supplement 3 video demonstrates the higher optical throughput achieved by the spectral multiplexing performed by the presented imager, in comparison to direct spectral measurements performed by a narrowband tunable filter. The high optical throughput of the presented imager provides several important benefits. The first benefit is a relatively high SNR when acquiring HS images in low photon-flux scenarios. This is an important advantage in airborne and spaceborne remote sensing tasks, where the exposure time for a single image is limited due to the fast relative motion between the imager and imaged object, and the desire to avoid capturing blurry images. The simulation demonstrated an approximately two orders of magnitude gain in optical throughput compared to the conventional push-broom avionic HS instrument, implying that the presented imager could capture HS images of objects that are approximately two orders of magnitude dimmer, if built with the same physical specifications. This significant gain in optical throughput still holds when comparing the presented imager to other HS imagers, which perform direct spectral measurements and no spectral multiplexing. According to the Nanosatellite & Cubesat Database^46^ the HyperScout^47^ and AaSI^44^ are the only HS nanosatellite imaging payloads with space heritage, which both directly measure the spectral attributes of the scene. This implies that the presented imager achieves state-of-the-art optical throughput values when compared to HS nanosatellite imaging payloads with space heritage. A second important benefit relates to the fact that the spectral irradiance intercepted by an imager is proportional to the square of its numerical aperture (NA)^48^. This means that the presented imager could be miniaturized so that its NA is significantly smaller than conventional HS imaging instruments, and still measure standard spectral irradiance values, as the high optical throughput compensates for the small NA. The imager could then be further miniaturized by attaching its 2D sensor array to the LCC (see Methods – Experimental optical setup) and reducing its physical volume. It should be mentioned that the spatial optical resolution is worsened when an imager’s aperture is reduced^48^ which for example limits the quality of drone and nano-satellite imaging.

As the presented imager performs passive along-track image acquisition, is of small size, has a high optical throughput and great spectral and spatial resolution, it is an excellent HS imaging payload for small airborne and sapceborne platforms. Additionally, although the presented camera performs along-track scanning, compared to the classical push-broom imaging method it collects spatial information of more than just one spatial line of the imaged scene at a time. This allows to prevent geometrical image distortions that are caused by system vibrations during acquisition, by using simple image registration algorithms. In classical push-broom cameras, correcting these distortions demands additional hardware^49,50^. Apart from the properties mentioned above, the presented camera has two limitations worth mentioning. The first limitation is the requirement of large processing resources for the spectral demultiplexing of the acquired HS images. However, the computational running time can be significantly reduced by performing parallel processing, as no spatial multiplexing is performed and each spatial pixel in the HS image can be independently spectrally demultiplexed. The computational running time could then be further shortened by performing the processing offline, on the ground, using high-end GPUs and multi-core CPU systems. A second limitation, which exists in conventional HS push-broom imagers as well, relates to capturing time varying scenes. If the scene changes within the acquisition time, that is, during the along-track scanning process, the final demultiplexed HS image will be distorted.

As HS data requires large computational storage resources, and as the presented imager performs varying spectral multiplexing, it is worth exploring the possibility to compress the spectral data which is acquired by the presented imager, using compressive sensing (CS)^51^ methods. Similar to the methods presented in^24,27,52–56^, the compression could be achieved by selecting a set of spectral filters which is compatible with the CS framework, for performing the spectral multiplexing during the along-track scanning process.

Apart from airborne and spaceborne remote sensing tasks, the presented imager can replace push-broom based HS imagers used in other applications, such as microscopy^57^ and in production lines^58^. Additionally, although we demonstrated the image acquisition in the visible spectral range, the imager could be adapted to other spectral ranges, making it relevant for many interesting tasks.

In conclusion, in this paper we have presented a design of a new camera, which is able to perform both HS and panchromatic imaging. The camera utilizes a wedge shaped LCC which allows it to passively acquire HS images by performing along-track scanning and spectral multiplexing. It therefore enjoys a significant gain in optical throughput when compared to classical HS imaging systems, and is tolerant to vibrations. Additionally, a panchromatic image can be acquired in a single snapshot when a voltage is applied to the LCC. An experimental optical setup of the imager was assembled, and its capabilities were successfully demonstrated in experiments and a simulation.

## Methods

### LCC device

The fabrication process of the wedge shaped LCC consists of several stages. At first, two flat glass plates are coated with Indium Tin Oxide (ITO) and a polyimide (NISSAN SE3510) alignment layer, forming electrodes that allow applying a voltage to the cell. The polyimide is spun on top of the ITO, baked and rubbed unidirectionally using a soft cloth. As a result, micro grooves are formed which are responsible for the alignment of the liquid crystal. Next, the plates are arranged in an anti-parallel geometry, and spacers of different sizes are applied at the corners to obtain a geometrical profile of a wedge. Glass spacers with a size of $1.9\mu m$ are applied at one edge, and small mylar sheet pieces of size $50\mu m$ at the other edge. Lastly, a nematic BL036 mixture (Merck) fills the gap by capillary action, and the LCC is placed between two linear crossed polarizers, oriented at an angle of 45 degrees with respect to the optical axis of the LCC. The obtained LCC has a clear aperture of about 8 mm × 8 mm.

### Experimental optical setup

**Figure 7:**
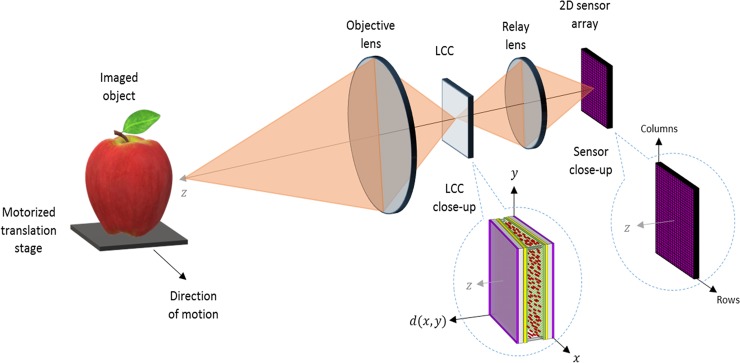
Experimental optical setup. The system is comprised of an objective lens which forms an image on the wedge shaped LCC device (described in Fig. 1). The image is then relayed to the 2D sensor array which is aligned with the LCC so that the ST varies only from column to column of pixels. The imaged object was placed on a motorized translation stage and moved in the direction of the sensor rows.

An illustration of the experimental optical setup of the presented imager is shown in Fig. 7. The objective lens forms an image on the wedge shaped LCC which is optically conjugated to the 2D sensor array using a 1:1 relay lens. Therefore, the image is firstly spectrally filtered according to the spatially varying ST of the LCC, and then captured by the 2D sensor array. The LCC and the 2D sensor array are aligned so that the LCC’s cell gap varies only along the sensor’s pixel rows and is approximately constant along the pixel columns. Therefore the ST, which depends on the value of the cell gap (Eq. 1), varies from column to column of pixels, but is identical at each row of pixels. This allows performing along-track scanning of a scene and spectrally encoding each of its imaged strips differently. It should be noted that the relay lens can be omitted if the sensor is attached to the LCC, thus reducing the system’s volume. The 2D sensor array used in the setup was a uEye CMOS UI-3240CP-C-HQ with 1280 $\times$ 1024 pixels, with a pixel size of 5.3 × 5.3 *μm* with 12-bit grayscale level radiometric sampling. Additionally, the setup consisted of a computer to control the image acquisition, and the voltage function generator which was used to drive a sine waveform voltage through the LCC for its panchromatic imaging mode. The relative motion between the imager and the imaged objects which was needed for the along-track scanning process, was achieved by placing the imaged objects upon a motorized translation stage, and emulating spaceborne and airborne imaging motion velocities by adjusting the camera’s exposure time. The stage was constructed in-house using an Arduino-Nano board and a step-motor, and was also controlled via the computer. The stage was aligned so that the objects were moved along the direction of the sensor’s rows.

### Calibration

**Figure 8:**
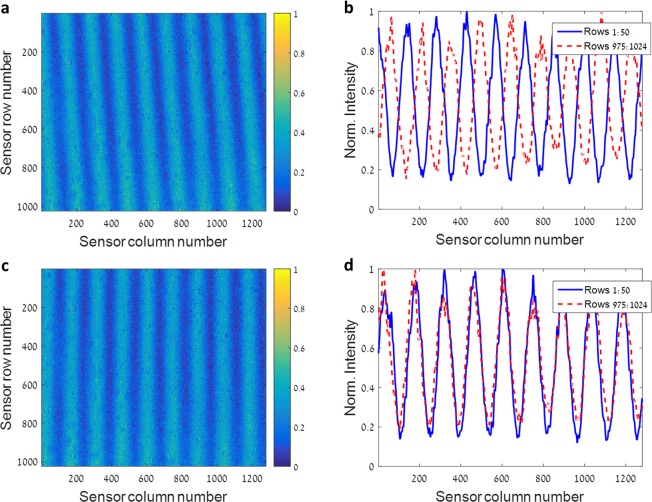
LCC and 2D sensor array alignment process. (**a**) The normalized intensity of the spatial cosine pattern measured by the 2D sensor array when the system is flooded with monochromatic light, and the LCC is not properly aligned with the sensor. (**b**) Comparison between the average normalized intensity measured at rows 1:50 (blue solid line curve) and 975:1024 (red dashed line) of (**a**). (**c**) The spatial cosine pattern obtained when the LCC and sensor are aligned. (**d**) Comparison between the average normalized intensity measured at rows 1:50 (blue solid line curve) and 975:1024 (red dashed line) of (**c**). The great agreement implies that the system is calibrated.

The calibration of the experimental optical setup is comprised of two stages. The goal of the first stage is to align the LCC with the 2D sensor array so that the cell gap varies only along the direction of the pixel rows, that is, from column to column of pixels. This is achieved by flooding the imager with monochromatic light using a halogen light source and a monochromator, followed by rotating the LCC in the image plane while observing the image obtained on the sensor plane. As the monochromatic input light is filtered by the oscillatory ST given in Eq. 1, a spatial cosine pattern appears on the sensor, oscillating in the direction of the varying LCC gap (Fig. 8a). From Eq. 1, one can observe that the frequency of the cosine pattern depends on the slope of the wedge, that is, the change in the cell gap, and on the wavelength of the monochromatic input light source. By rotating the LCC, the spatial cosine pattern can be aligned with the sensor’s rows of pixels, obtaining identical cosine waveforms in each row. Figure 8 demonstrates the alignment process. Figure 8a shows an unaligned spatial cosine pattern on the sensor array, and Fig. 8b shows the resulting difference between the averaged cosine pattern obtained in two different ranges of rows of pixels, one at the top and one at the bottom of the sensor. Figure 8c shows the aligned spatial cosine pattern, and Fig. 8d shows the averaged cosine patterns of the two ranges of rows of pixels used in Fig. 8b, which are in great agreement after the alignment is complete. For this stage of the calibration process, the wavelength of the monochromatic input light used was 639 nm.

The second stage of the calibration process focuses on obtaining a full spectral-spatial characterization of the ST of the LCC and the entire system. This stage is crucial as the ST is a required input for the solvers mentioned in the Results section, which are used for the spectral demultiplexing process of the acquired HS images. As the effective birefringence function introduced in Eq. 1 is unknown, the theoretical ST expression (Eq. 1) cannot be used directly and the ST must be experimentally measured. One optional method to obtain the desired characterization of the ST, which is explained in^36,59^, is to measure the ST at several locations on the LCC using a moveable point light source, and estimate it at all other locations. In this work, we applied an alternative method. Using the same halogen light source and monochromator from the previous calibration stage, the imager was flooded with monochromatic light which was scanned from 450 *μm* to 700 *μm*. An image of the spatial cosine pattern was captured per scanned wavelength, and the images were stacked to form a HS data-cube. The captured data-cube has two spatial dimensions, corresponding to the rows and columns of the sensor, and one spectral dimension, with each spectral channel corresponding to a specific scanned wavelength. By measuring the spectral power density of the halogen light source using a grating based spectrometer, and by using the captured data-cube, the normalized ST was calculated per spatial pixel. As the LCC and the sensor were previously aligned, the ST was identical along the pixel rows. Therefore, the ST of the pixel rows was averaged to obtain better SNR in its characterization. Because the full ST characterization is needed for the spectral demultiplexing of the acquired HS images, the second stage of the calibration process was performed with no voltage applied to the LCC, and its results were previously shown in Fig. 1b. It should be noted that the ST map shown in Fig. 1c, which demonstrates that when a voltage is applied to the LCC, a ST suitable for panchromatic imaging is obtained, can be measured in a similar manner while driving a voltage through the LCC.

## Supplementary information

Supplement 1.

Supplement 2.

Supplement 3.
